# *Toxoplasma gondii* suppress human cord blood cell differentiation to the NK cell population

**DOI:** 10.1002/iid3.1329

**Published:** 2024-06-21

**Authors:** Sepideh Mahmoudzadeh, Khadijeh Dizaji Asl, Hojjatollah Nozad Charoudeh, Reza Rahbarghazi, Mahdi Ahmadi, Morteza Heidarzadeh, Adel Spotin, Ehsan Ahmadpour

**Affiliations:** ^1^ Immunology Research Center Tabriz University of Medical Sciences Tabriz Iran; ^2^ Department of Parasitology and Mycology, Faculty of Medicine Tabriz University of Medical Sciences Tabriz Iran; ^3^ Department of Histopathology and Anatomy, Faculty of Medical Sciences, Tabriz Medical Sciences Islamic Azad University Tabriz Iran; ^4^ Department of Applied Cell Sciences, Stem Cell Research Center Tabriz University of Medical Sciences Tabriz Iran; ^5^ Drug Applied Research Center Tabriz University of Medical Sciences Tabriz Iran; ^6^ Sarab Faculty of Medicine Sarab Iran; ^7^ Koç University Research Center for Translational Medicine (KUTTAM) Istanbul Turkey

**Keywords:** differentiation, functional activity, maturation, NK cells, *Toxoplasma gondii*, umbilical cord blood mononuclear cells

## Abstract

**Background:**

*Toxoplasma gondii* is an obligate intracellular protozoan parasite that can invade all mammalian cells. It is well established that natural killer (NK) cells have critical protective roles in innate immunity during infections by intracellular pathogens. In the current study, we conducted an in vitro experiment to evaluate NK cell differentiation and activation from human umbilical cord blood mononuclear cells (UCB‐MNCs) after infection with *T. gondii* tachyzoites.

**Methods:**

UCB‐MNCs were infected by fresh tachyzoites of type I (RH) or type II (PTG) strains of *T. gondii* pre‐expanded in mesenchymal stem cells for 2 weeks in a medium enriched with stem cell factor, Flt3, IL‐2, and IL‐15. Flow cytometry analysis and western blot analysis were performed to measure the CD57^+^, CD56^+^, and Granzyme A (GZMA).

**Results:**

Data revealed that incubation of UCB‐MNCs with NK cell differentiation medium increased the CD57^+^, CD56^+^, and GZMA. UCB‐MNCs cocultured with PTG tachyzoites showed a significant reduction of CD56^+^ and GZMA, but nonsignificant changes, in the levels of CD56^+^ compared to the control UCB‐MNCs (*p* > .05). Noteworthy, 2‐week culture of UCB‐MNCs with type I (RH) tachyzoites significantly suppressed CD57^+^, CD56^+^, and GZMA, showing reduction of NK cell differentiation from cord blood cells.

**Conclusion:**

Our findings suggest that virulent *T. gondii* tachyzoites with cytopathic effects inhibit NK cell activation and eliminate innate immune responses during infection, and consequently enable the parasite to continue its survival in the host body.

## Graphical abstract

The study investigated the impact of *Toxoplasma gondii* on natural killer (NK) cell activation and differentiation from human umbilical cord blood mononuclear cells. It found that exposure to *T. gondii* tachyzoites led to reduced activation and differentiation of NK cells, particularly with the virulent RH strain suppressing NK cell markers. These findings suggest that *T. gondii's* effect on NK cells may allow the parasite to evade the host's innate immune response and persist in the body.

## 1 INTRODUCTION

*Toxoplasma* *gondii* is a protozoan apicomplexan parasite that affects about 30% of the human population. It was suggested that this protozoan potentially infects all mammalian and avian nucleated cells via vertical and horizontal transmissions. The acute form of toxoplasmosis is asymptomatic in healthy individuals. However, the onset of toxoplasmosis in immunocompromised individuals (i.e., people with HIV/AIDS) can cause severe neurological and ocular complications with congenital abnormalities in developing fetuses.^1^, ^2^, ^3^ Relevant to the developmental profile, *T. gondii* is initially found in the intestines after oral ingestion followed by passing across the physiological barriers and expansion to the lymphoid tissues and reticuloendothelial system. In the latter stages, several tissues such as muscles, myocardium, central nervous system especially brain parenchyma can be infected.^4^, ^5^, ^6^

Among different immune cell types, natural killer (NK) cells with phenotypic markers of CD3^−^CD56^+^CD16^±^ are the predominant innate lymphocyte subsets belonging to the group 1 innate lymphoid cells family. These cells are mainly involved in the recognition and destruction of anaplastic cells, as well as combating intracellular pathogen infections caused by viruses, bacteria, and parasites.^2^, ^7^, ^8^, ^9^ NK cells eliminate pathogen‐harbored cells by releasing a plethora of proinflammatory cytokines, including interferon‐gamma (IFN‐γ), tumor necrosis factor‐alpha/beta (TNF‐α/β), CD95/FasL, and TNF‐related apoptosis‐inducing ligand (TRAIL), as well as cytoplasmic cytotoxic granules containing pore‐forming perforin (PFN), granulysin (GNLY), and granzymes A (GZMA) and B a.^10^, ^11^, ^12^, ^13^ It has been found that the early stages of toxoplasmosis (acute infection) can provoke NK cells, which are touted as frontline innate immunity.^2^, ^14^, ^15^ CD57 is integral to a functionally distinct population of mature NK cells in the human CD56^dim^CD16^+^ NK‐cell subset. It has been reported that the expression of CD57 is associated with NK cell maturation and phenotypic acquisition during chronic infections.^6^, ^16^ In the current study, we have evaluated CD57^+^, CD56^+^, and GZMA expressions as markers of NK cell stimulation in response to *T. gondii* infection.

## 2 MATERIALS AND METHODS

### 2.1 Expansion of Type I and II strains of *T. gondii*

Tachyzoites of Type I (RH) and II (PTG) strains of *T. gondii* were provided by the Toxoplasmosis Research Center at Mazandaran University of Medical Sciences. To obtain freshly viable RH tachyzoites, the parasite was maintained through serial passage and intraperitoneal inoculation. Female BALB/c mice (8−12 weeks old) were intraperitoneally infected with 2 × 10^4^ tachyzoites. Four days later, tachyzoites were harvested from the peritoneum of the infected mice.

Regarding the PTG strain, the infected mice were euthanized, and the mouse brains were harvested, washed with PBS, and placed inside microtubes. Using a syringe, the brain tissue was repeatedly aspirated and expelled until a completely homogenized brain sample was obtained. The resulting suspension contained cysts of the *T. gondii* PTG strain, which were reinjected into mice for maintenance and passaging. The cysts reformed in the infected mice over a period of approximately 2−3 months. The inoculated mice were daily monitored for up to 2 months. Ethical approval was obtained from the Ethics Committee of Tabriz University of Medical Sciences (IR.TBZMED.REC.1398.085).

### 2.2 Bone marrow mesenchymal stem cells (BM‐MSCs) culture protocol

In this study, human BM‐MSCs were used as an intermediate cell to obtain pure tachyzoites devoid of potential contaminations or additional antigens. These cells are easily expandable with immunosuppressive properties. Commensurate with these comments, BM‐MSCs can be used as suitable cell sources for the expansion of *T. gondii* culturing.^17^, ^18^ Human BM‐MSCs were obtained from the Stem Cell Research Center (Tabriz University of Medical Sciences). Cells were cultured in low‐glucose‐content DMEM (DMEM/LG) medium under standard conditions (95% relative humidity at 37°C with 5% CO_2_). The culture medium was changed every 3−4 days. Upon 70%−80% confluence, BM‐MSCs were passaged using 0.25% Trypsin‐EDTA solution. Cells at passage 3 were used for analyses.

### 2.3 Infection of BM‐MSCs with *T. gondii* tachyzoites

To this end, different tachyzoite to BM‐MSC ratios, including 1:1, 1:10, 1:100, and 1:1000 were used. For each ratio, BM‐MSCs were separately infected with RH/PTG strains in culture flasks for 2 weeks. During the culture period, the pH value was carefully monitored every 24 h. We found that 2‐week culture of BM‐MSCs with RH/PTG strains led to intracellular proliferation. In the next step, cells were passaged and the number of viable tachyzoites using the Neubauer counting chamber. The procedure was continued with the culture of BM‐MSCs in 96‐well plates for the next 24 h.

### 2.4 Isolation of cord blood mononuclear cells (MNCs)

Umbilical cord blood (UCB) samples were collected from full‐term normal deliveries at the Shahid Taleghani Hospital. The volunteers were asked to complete the informed consent. All procedures of this study were confirmed by the Local Ethics Committee of Tabriz University of Medical Sciences. In brief, samples were collected in a 50 mL heparinized Falcon tube and immediately transported on dry ice to the laboratory. UCB samples were diluted 1:2 with PBS supplemented with 5% fetal bovine serum (FBS; Gibco). To isolate UCB‐MNCs, the diluted samples were gently overlaid on Ficoll‐Hypaque (Sigma‐Aldrich) and centrifuged at 400×*g* for 25 min at 4°C. UCB‐MNCs were carefully collected at the interphase and washed twice with PBS. Cells were resuspended in RPMI 1640 (Gibco) supplemented with 10% FBS. The viability of UCB‐MNCs was determined by using 0.4% Trypan blue staining. UCB‐MNCs were incubated with tachyzoite at a ratio of 1:1000 in each well of 96‐well plates.

To assess the stimulatory effect of *T. gondii* tachyzoites on the activation of NK cells, UCB‐MNCs, and infected BM‐MSCs were cocultured at the density of 1 × 10^5^ cells per well of 96‐well plates in using 200 μL culture medium enriched with 10% FBS, 1% penicillin/streptomycin (Sigma‐Aldrich), and a cocktail of cytokines (stem cell factor, Fms‐like tyrosine kinase 3 [Flt3], interleukin‐2 [IL‐2], and interleukin‐15 [IL‐15]; eBioscience). The final concentration of all cytokines reached 50 ng/mL. Cells were cultured for 7 days.

### 2.5 Flow cytometry analysis

On Day 14, flow cytometry analysis was performed using antibody targeting surface CD57 (PE/CY5, UCHT1; BioLegend). In short, cells were collected and washed twice with PBS containing 5% FBS. Next, the antibody was added to 50 μL cell suspension and incubated for 20 min at 4°C. Using 7‐amino‐actinomycin D (7ADD, 0.25 μg/108 cells; BD Bioscience), dead cells were excluded during the analysis. About 10,000−30,000 events were analyzed for each sample in the FACSCalibur system (BD eBioscience) and FlowJo (7.6.1) software.

### 2.6 Western blot analysis

Western blot analysis was done to measure protein levels of CD56 and GZMA after treatment with RH and PTG strains of *T. gondii.* For this purpose, cells were centrifuged at 12,000 rpm for 10 min at 4°C, the collected pellets washed twice with cold PBS and lysed using RIPA lysis buffer and protease inhibitors. Total protein content was measured by Bradford assay (Bio‐Rad). Samples were separated on 12% SDS‐PAGE and electro‐transferred onto a polyvinylidene difluoride (PVDF) membrane (GE Healthcare). The membranes were blocked in 2% skim milk dissolved in TBS‐T (20 mM Tris, 137 mM NaCl, and 0.1% Tween 20) for 1 h. The membranes were incubated with primary monoclonal antibodies against β‐actin (dilution: 1:300) and CD56 (dilution: 1:200, Cat no: sc‐7326; Santa Cruz) and GZMA (1:200, Cat no: sc‐33692; Santa Cruz) at 4°C for 18 h. Following three‐time washes in TBS‐T, the membranes were incubated with goat anti‐mouse secondary antibody (dilution: 1:1000, Cat no: sc‐2357; Santa Cruz) for 1 h. Reactive immunoblots were visualized using a chemiluminescence Kit (ECL; GERPN418) and X‐ray film (Fujifilm). The intensity of protein bands was normalized by the corresponding β‐actin using ImageJ 1.6 software (National Institute of Health).

### 2.7 Statistical analysis

**Figure 1:**
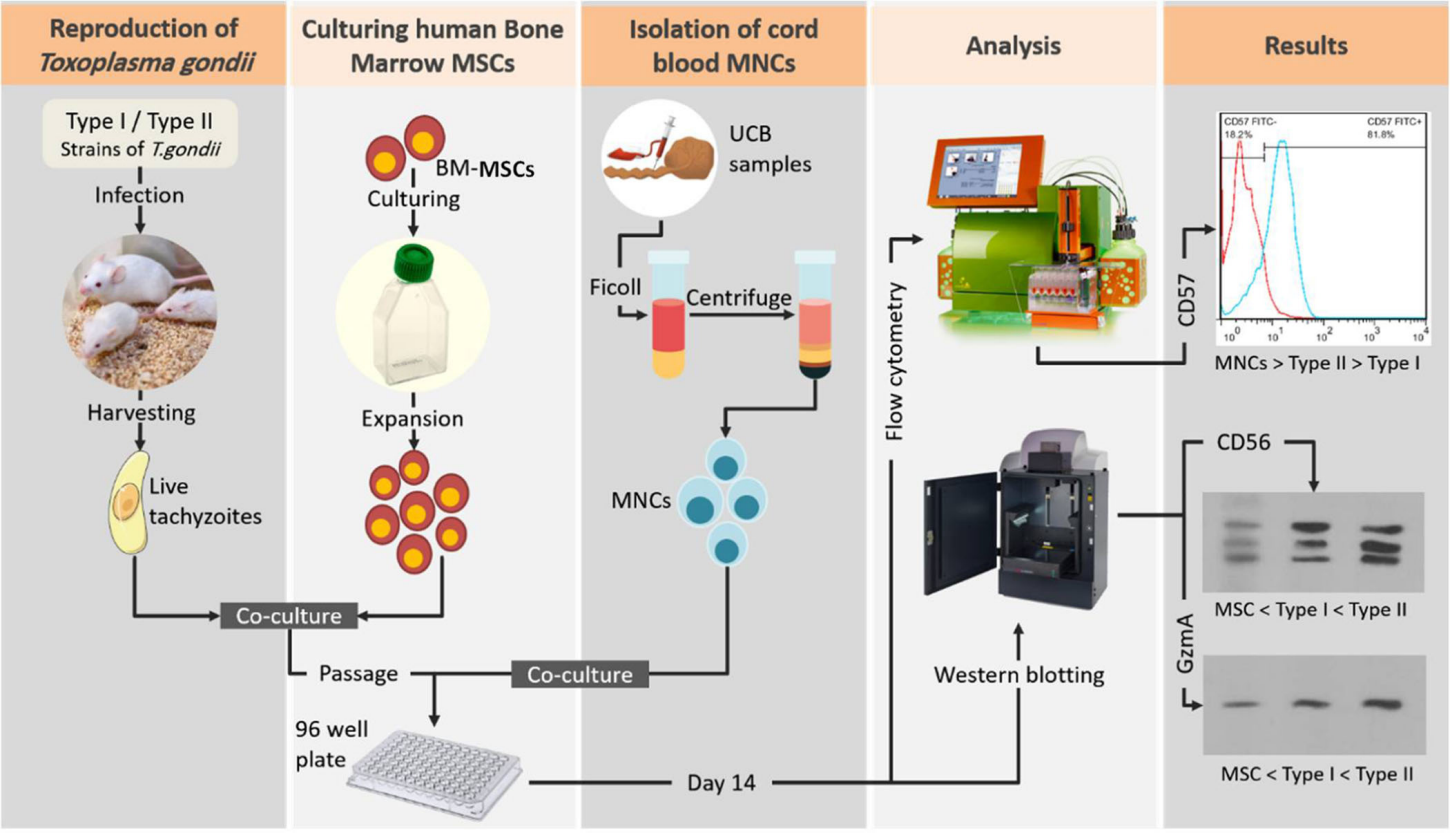
Overview of the experimental approach and the main findings of the study. BM, bone marrow; MNC, mononuclear cells; UCB, umbilical cord blood.

Statistical analyses were performed using GraphPad Prism version 6.01 (GraphPad Software Inc.). All data were expressed as the means ± SD by triplicate independent experiments. The statistical analysis of differences was performed using one‐way and two‐way analysis of variance followed by Tukey's multiple comparisons test and Bonferroni's multiple comparisons test. The *p* < .05 was considered to indicate a statistically significant difference. Figure 1 provides an overview of the experimental approach and the main findings of the study.

## 3 RESULTS

### 3.1 BM‐MSCs culture and expansion

**Figure 2:**
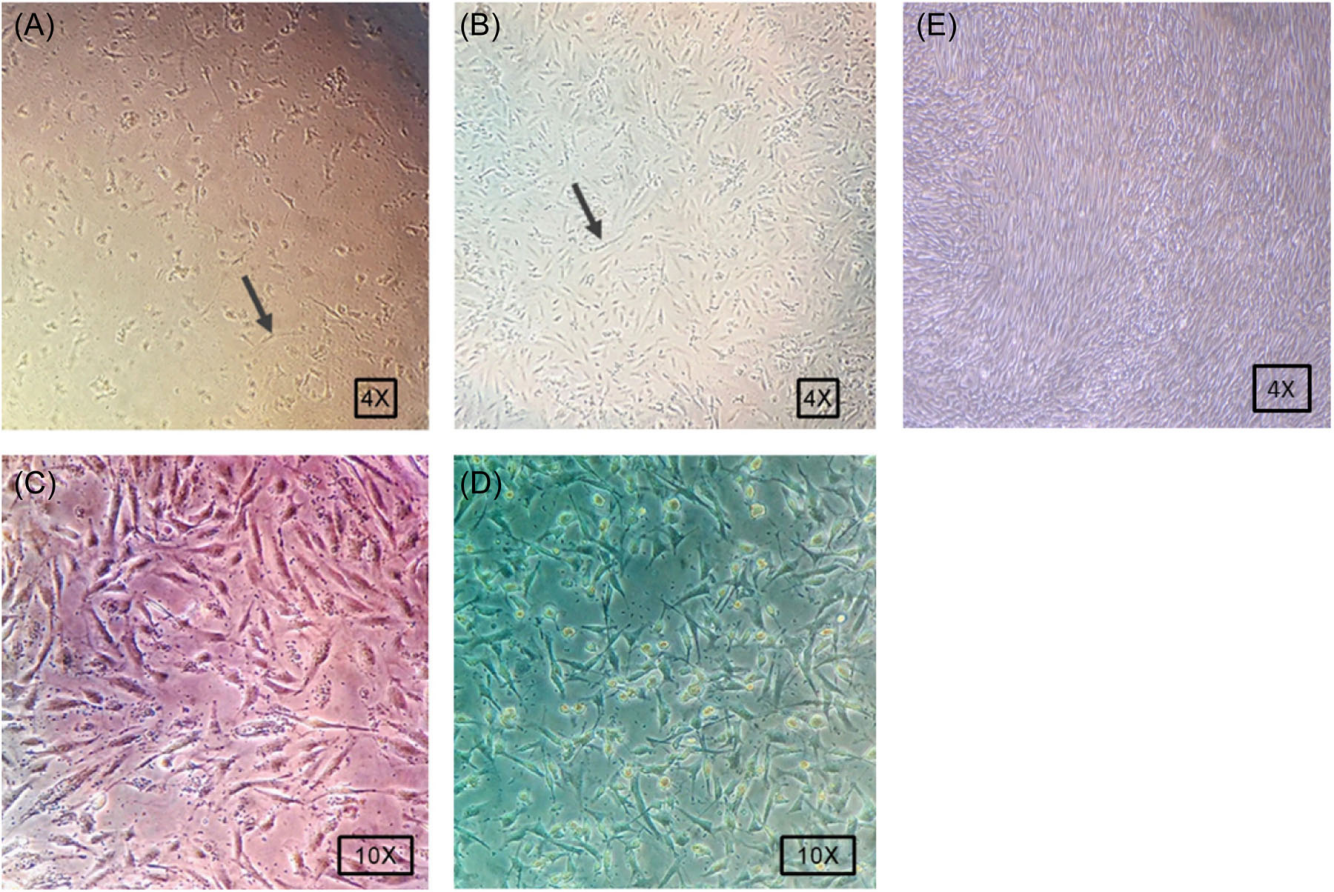
Incubation of human BM‐MSCs with *Toxoplasma* *gondii* tachyzoites at different ratios (A) 1:10 and (B) 1:100 for 2 weeks (arrow points to BM‐MSCs). By increasing tachyzoites numbers, BM‐MSCs exhibited cytotoxic indicated with the reduction of cell number per cm^2^ and loss of spindle shape morphology. Incubation of human BM‐MSCs with PTG (C) and RH (D) *T. gondii* tachyzoites for 2 weeks. (E) Control noninfected MSCs. BM‐MSCs, bone marrow mesenchymal stem cells.

In this study, we used human BM‐MSCs as an intermediate cell source for the expansion of *T. gondii* tachyzoites. Bright‐field imaging displayed spindle‐shaped fibroblast‐like cells 2−3 days after initial plating. On Day 7, BM‐MSCs formed relatively a monolayer confluent cell with a whirling appearance. These data showed the efficiency of our protocol in the isolation and expansion of human BM‐MSCs (Figure 2).

### 3.2 *T. gondii* tachyzoites decreased human BM‐MSCs in a dose‐dependent manner

Forty‐eight hours after the incubation of human BM‐MSCs with tachyzoites, about 95% of cells were infected (Figure 2). It was suggested that in a coculture system with tachyzoite to BM‐MSC ratios of 1:1, 1:10, and 1:100, a large number of cells (more than 50% BM‐MSCs) died. The cells lost spindle shape and acquired a round form, showing the attachment removal. This effect is possibly associated with the accumulated number of intracellular tachyzoites inside the cells. As a correlate, further analyses were done in a group with ratios of 1:1000. According to our data, incubation of human BM‐MSCs with RH tachyzoites (1:1000) yielded a low cell survival rate compared to the group that received PTG strain (Figure 2).

### 3.3 *T. gondii* tachyzoites can reduce the orientation of UCB‐MNCs toward CD57^+^ NK cells

**Figure 3:**
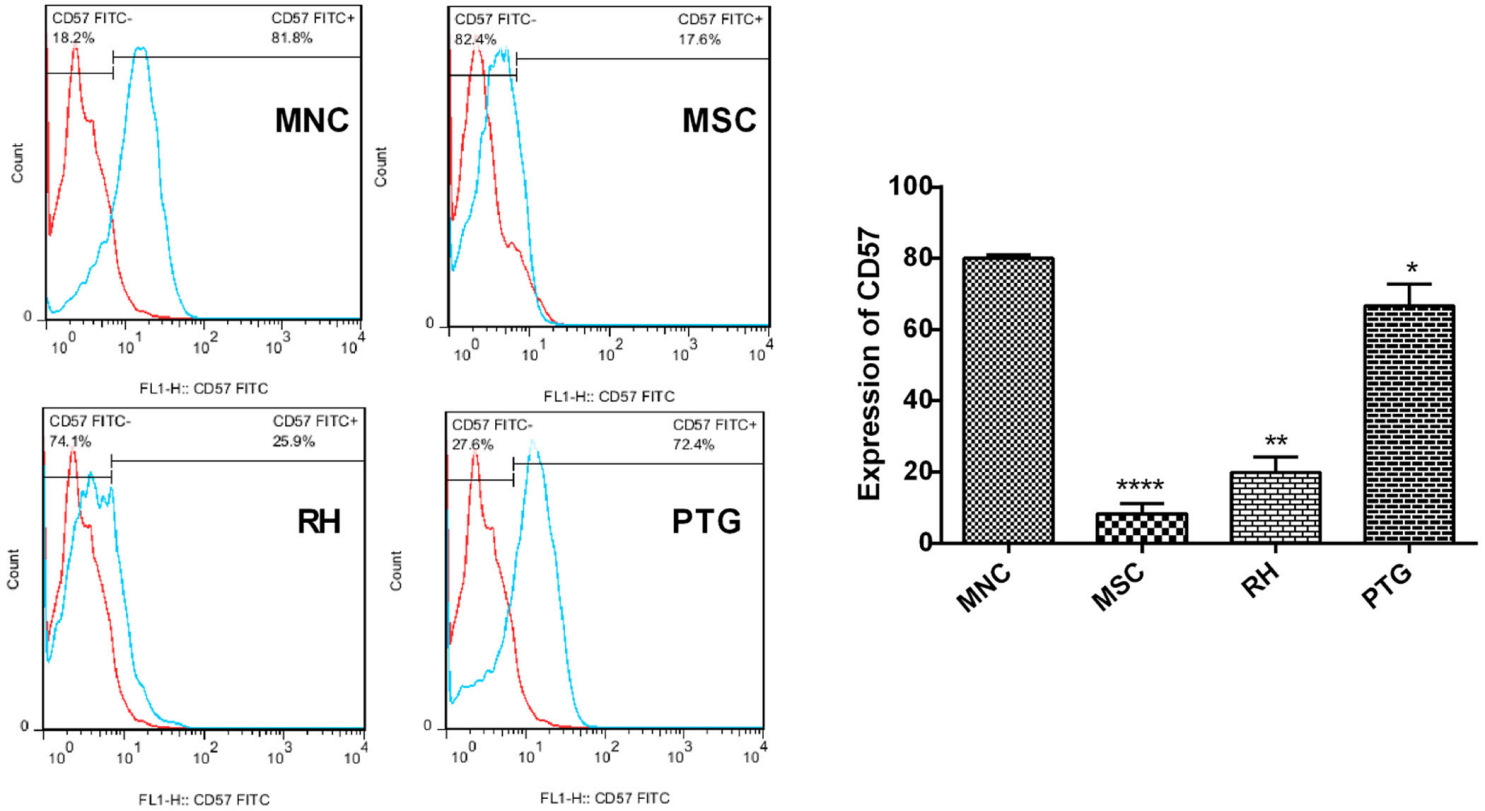
CD57^+^ expression was evaluated on infected UCB‐MNCs using flow cytometry analysis after 14 days. In the MNC group, the highest expression of CD57^+^ was achieved (left). The expression of CD57^+^ on UCB‐MNCs exposed to PTG stain was high compared to the group that received the RH strain. The lowest CD57^+^ expression rate was obtained in human BM‐MSCs after exposure to tachyzoites (right). All experiments were performed in triplicate. Significant differences were observed between the MNC group and other groups (*p < .05; **p < .01, ****p < .0001). BM‐MSCs, bone marrow mesenchymal stem cells; UCB‐MNCs, umbilical cord blood mononuclear cells.

We evaluated the expression of CD57^+^ NK cells in UCB‐MNCs using flow cytometry analysis after 14 days (Figure 3). We noted that the percent of CD57^+^ cells reached 81.8% in the control UCB‐MNCs. According to our data, these values were reduced to 72.4% in cells exposed for PTG tachyzoites (*p* < .05). Also, data showed a significant reduction of CD57^+^ cells in the group exposed to RH tachyzoites compared to the control UCB‐MSCs (*p* < .01). Of note, MSCs cocultured with tachyzoites exhibited less capacity to differentiate into the CD57^+^ cells. These data showed that incubation of UCB‐MNCs with RH tachyzoites can prohibit differentiation capacity into NK cells (Figure 3).

### 3.4 *T. gondii* tachyzoites diminished GZMA and CD56 synthesis in UCB‐MNCs

**Figure 4:**
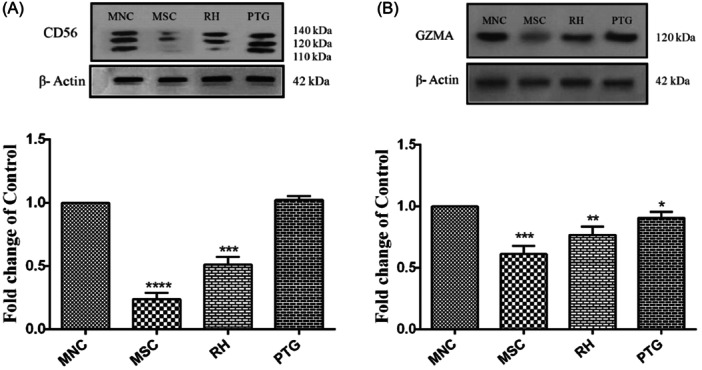
Measuring protein levels of CD56 (A) and GZMA (B) in UCB‐MNCs after exposure to RH and PTG *Toxoplasma gondii* tachyzoites. The results are shown as the mean ± SD of three independent experiments. There was a statistically significant difference in MNC and other groups (*p < .05; **p < .01, ***p < .001, ****p < .0001). GZMA, granzyme A; UCB‐MNCs, umbilical cord blood mononuclear cells.

To study the possible effects of *T. gondii* infection on the differentiation of UCB‐MNCs into NK cells and functional activity, we measured protein levels of CD56 and GZMA expressions, respectively, 14 days after the incubation with tachyzoites. We found that exposure of UCB‐MNCs with tachyzoites led to a decrease of CD56 and GZMA compared to the MNC noninfected control group. We found that the presence of highly pathogenic *T. gondii* tachyzoites (RH strain) abrogated these effects, indicating a decrease in NK cell differentiation induced by UCB‐MNCs (Figure 4).

## 4 DISCUSSION

NK cells have a critical role in innate immunity and the destruction of infected cells by cytotoxicity.^2^, ^5^ These cells can participate in the early resistance responses against parasitic infections such as *T. gondii*.^19^ Within hours after infection with *T. gondii*, NK cells release distinct cytokines IFN‐γ and TNF‐α that limit the further expansion of parasites from primary sites to other places.^7^ Depending on the activation state, NK cells invariably express CD2, CD16, CD56, and CD57 surface markers. Based on a panel of surface markers, NK cells can be conveniently isolated into subsets for functional analyses.^20^ In this study, we investigated the cellular distribution of CD57, CD56, and GZMA as markers of the activation and differentiation of NK cells using an in vitro culture system in UCB‐MNCs after incubation with RH and PTG strains of *T. gondii*. Results showed that UCB‐MNCs act differently after incubation with two different strains of *T. gondii*. To be specific, PTG tachyzoites did not significantly alter the survival rate of UCB‐MNCs after 2 weeks, while the coculture of these cells with highly virulent tachyzoites, RH reduced viability compared to the noninfected control group. It is postulated that the presence of virulent tachyzoites not only prohibits NK cell differentiation of human UCB‐MNCs but also promotes remarkable cytopathic effects on these cells. One potential explanation for this observation is the notably higher pathogenicity associated with the RH strain in contrast to the PTG strain. In accordance with current data, Robben et al. indicated PTG tachyzoites can efficiently stimulate the production of proinflammatory cytokines and particularly high levels of the Th1‐polarizing cytokine such as IL‐12. By contrast, in the presence of RH tachyzoites, trivial levels of proinflammatory cytokines are detectable.^21^ The precise mechanisms supporting these effects are the subject of debate. It has shown that IL‐12 is a pivotal cytokine for the generation of IFNγ–producing NK cells in line with the maturation of CD4^+^ and CD8^+^ lymphocytes which are critical for adaptive immunity against parasites.^22^ Giving to our statistics, incubation of human BM‐MSCs with RH tachyzoites (1:1000) yielded a low cell viability rate rather than to the group that received PTG strain.

The role of CD56 on NK cell activation is mostly unknown, but published data illustrate a close relationship between the intensity of surface marker CD56 and the degree of activation.^23^ In support of this notion, low levels of CD56 on MNCs were detected after infection with Type I strain (RH) of *T. gondii* tachyzoites. Like *Toxoplasma*, *Leishmania* (*L.*) promastigotes can reduce CD56 expression and protein levels in NK cells. While the decrease in CD56 mRNA was comparable for *L. major*, *L. infantum*, and *L. mexicana*, surface CD56 was less strongly downregulated by *L. major* as compared with *L. infantum* and *L. mexicana*.^24^, ^25^ Besides, Hermann et al. found that the proportion of CD56^bright^ NK cells was significantly decreased in cord blood from newborns congenitally infected with *Trypanosoma cruzi*.^26^ Whether and how CD56 participates in NK cell activity needs further investigation. Very recent data support the crucial role of CD56 in NK cell maturation and migration.^27^ Pathogenic parasitic infections are accompanied by the emergence of a large fraction of cells expressing CD57^+^, a marker related to highly cytotoxic CD8^+^ or NK cells.^28^ CD8^+^ T cells play a main role as effector lymphocytes against *T. gondii* whereas CD4^+^ T cells are crucial for the regulation of the immune response against *T. gondii*.^29^ Garcia‐Munoz et al. reported an expansion of CD8^+^CD57^+^ T cells in young patients with an acute infection of *T. gondii* indicating the importance of CD8^+^CD57^+^ T cells in the control of the chronic phase of intracellular microorganisms.^30^ Data suggested the presence of NK, CD57^+^, CD4^+^, and CD8^+^ T cells, CD20^+^ B cells, as well as CD68^+^ macrophages in biopsies prepared from patients with ulcerated cutaneous *L.* (CL) lesions. Noteworthy, the number of CD57^+^ cells closely correlates with macrophages and amastigotes. These features support the possible participation of CD57^+^ cells in killing infected macrophages.^31^

The perforin/GZM‐mediated action of cytotoxic lymphocytes has been shown to be involved in host defense against a large number of intracellular pathogens, including *T. gondii*,^32^ and Granzymes represent a suitable marker for activation and degranulation of NK cells.^33^ Of note, plasma concentrations of GZMA and GZMB are meaningfully increased in patients with clinical *P. falciparum* in (semi)‐immune children, showing that NK cells are likely involved in innate immune human host resistance in the early phase of malaria infection.^34^ Likely, higher frequencies of NK cells are obvious in the peripheral blood of patients with CL as compared to the healthy subjects. Unlike the normal NK cells, the activated NK cells possess higher contents of GZMB and perforin than that of CD8^+^ T cells.^35^ Morphological investigation revealed that cytotoxic granules harboring Granzymes, perforin, and GNLY work together in a tight manner to kill three protozoan parasites, including *T. cruzi*, *T. gondii,* and *L. major*. PFN and GNLY disrupt host cell and parasite membranes, respectively, to bring the Granzymes into intracellular parasites, where they proteolytically act to generate reactive oxygen species and dismantle parasite oxidative defenses.^9^ In accordance with these results, our observation of increased production of GZMA in NK cells suggests that these cells could act rapidly during infection by the Type II (PTG) strain of *T. gondii* tachyzoites. The observed decrease in GZMA in MSCs may be attributed to the presence of cells that may be viable but nonfunctional.

Frequent studies have been conducted in the field of differentiation and the effect of NK cells in various parasitic infections, which indicated the vital role of these cell lines, cytokines, and their regulatory factors throughout different stages of both helminthic and protozoan infections. In the study by Roland et al., the contribution of NK cells in the immune response to primary infection with *Plasmodium yoelii* sporozoites in C57BL/6 mice was investigated.^36^ The study found that hepatic and splenic NK cells are activated during infection and exhibit different phenotypic and functional properties. Specifically, the number of hepatic NK cells increased, whereas the number of splenic NK cells decreased. In both the liver and spleen, NK cells showed activation during infection, as evidenced by the increased expression of the CD69 surface marker. Consistent with previous studies, CD25 molecules were not detected on the surface of naive or activated NK cells in either the liver or spleen during *P. yoelii* infection.

A study utilizing confocal and parabiosis microscopy has highlighted the significant role of NK cells in parasitic infections.^37^ However, ongoing research aims to elucidate the precise roles and mechanisms of NK cells in parasitic infections. Recent data on the interaction between parasitic organisms and NK cells, such as the study by Alizadeh et al., revealed the effect of *Leishmania* LPG on the NK cell population.^38^ They found that *Leishmania* LPG can limit NK cell activation by inhibiting IL‐12 synthesis in macrophages and that GP63 directly binds to NK cells and modulates their phenotype.

Goodier et al. provided evidence of direct interaction between malaria‐infected host cells and NK cells.^39^ They investigated how innate inflammatory signals induced by malaria parasite‐associated molecular patterns influence the indirect activation and function of NK cells. Furthermore, they found that anti‐malarial immunity advances in parallel with NK cell differentiation, marked by a reduced dependence on inflammatory signals and an enhanced ability of NK cells to target malaria parasites more precisely, particularly through antibody‐dependent mechanisms.

## 5 CONCLUSION

This study aimed to investigate NK cell differentiation of human UCB‐MNCs infected with PTG and RH strains of *T. gondii* tachyzoites. Data revealed an inverse correlation between CD57^+^, CD56^+^, and GZMA levels and the degree of parasite pathogenicity. Our data was showed that virulent *T. gondii* tachyzoites with cytopathic effects inhibit NK cell activation and eliminate innate immune responses during infection, thereby enabling the parasite to continue its survival in the host body.

## AUTHOR CONTRIBUTIONS

**Ehsan Ahmadpour, Hojjatollah Nozad Charoudeh, Reza Rahbarghazi, Adel Spotin, and Mahdi Ahmadi**: Conceptualization. **Sepideh Mahmoudzadeh, Khadijeh Dizaji Asl, Mahdi Ahmadi, and Morteza Heidarzadeh**: Methodology. **Ehsan Ahmadpour, Hojjatollah Nozad Charoudeh, Reza Rahbarghazi, and Mahdi Ahmadi**: Validation. **Sepideh Mahmoudzadeh, Khadijeh Dizaji Asl, Mahdi Ahmadi, and Morteza Heidarzadeh**: Investigation. **Ehsan Ahmadpour, Adel Spotin, and Hojjatollah Nozad Charoudeh**: Data curation. **Sepideh Mahmoudzadeh, Khadijeh Dizaji Asl, Mahdi Ahmadi, and Morteza Heidarzadeh**: Writing—original draft preparation. **Ehsan Ahmadpour, Hojjatollah Nozad Charoudeh, Adel Spotin, and Reza Rahbarghazi**: Writing—review and editing. All authors have read and agreed to the published version of the manuscript.

## CONFLICT OF INTEREST STATEMENT

The authors declare no conflict of interest.

## ETHICS STATEMENT

All experiments were approved by the local Ethics Committee of Tabriz University of Medical Sciences, Tabriz, Iran (No. IR.TBZMED.REC.1398.085).

## Data Availability

All data appeared in the submitted manuscript.
